# The development of the social isolation risk scale (SIRS) to identify risk for social isolation in head and neck cancer survivors

**DOI:** 10.3389/fragi.2026.1805909

**Published:** 2026-04-17

**Authors:** Allison Marziliano, Nicholas R. Eaton, Mahdia Parker, Maged Ghaly, Diana Schettini, Maureen Balram, Carla Perissinotto, Michael A. Diefenbach

**Affiliations:** 1 Northwell Health, New York, NY, United States; 2 Stony Brook University, Stony Brook, NY, United States; 3 University of California San Francisco, San Francisco, CA, United States

**Keywords:** Head and neak cancer, risk, screening, social science research, social isolation

## Abstract

**Introduction:**

The purpose of this manuscript is to report on the development of the SIRS (Social Isolation Risk Scale), a brief screening tool to identify HNC survivors at risk for social isolation through assessing for the presence of multiple potential causes from which this risk might arise.

**Method:**

The development of the SIRS occurred in two phases. During the first phase, item generation, the research team leveraged inductive and deductive methods to generate an exhaustive pool of potential scale items based on those in the extent literature and on the opinions of experts in HNC. During the second phase of development, item refinement, we conducted one-to-one interviews with 22 socially isolated HNC survivors. During analyses, items endorsed by 7 or more participants on the checklist were retained. Retained items were reorganized from most impactful to least impactful. General items (i.e., those concerns that were not specific to only HNC survivors, e.g., chemotherapy fatigue) were removed. The remaining items were reorganized so that items with similar content were placed together.

**Results:**

The item generation phase yielded 39 potential items for the SIRS. These 39 items were reviewed during the item refinement phase by a sample of N = 22 HNC survivors. On average, they were 66.41 years old (SD = 9.04 years), and mostly male (n = 17, 77%). Participants were an average of 3.18 years (SD = 4.81 years) since completing treatment. Of the 39 items, 29 were endorsed by 7 or more HNC survivors and retained. Of the list of 29 items, 16 additional items were removed, as they were not specific to HNC. The remaining 13 items, which were reorganized so that items of similar content were adjacent to each other, composed the SIRS. The stem questions and the Likert-scale response options used during the item refinement phase were retained for the final SIRS.

**Conclusion:**

As a first of its kind, SIRS is a brief screening tool for use in busy clinical environments to characterize risk for social isolation in HNC survivors. It was developed through rigorous psychometric methodology including both item generation and refinement phases.

## Introduction

Head and neck cancer (HNC), which encompasses several different malignant tumors that develop in and around the throat, larynx, nose, sinuses, mouth, and oropharynx, is the seventh most common cancer in the world, accounting for more than 660,000 new cases globally each year (Gormley et al., 2022). HNC disproportionately affects older adults, with about 60%–70% of cases diagnosed in patients over age 60–65 years, and up to 40% in those over 70 years. The incidence of HNC is increasing and is expected to continue to rise around the world (Gormley et al., 2022). Incidence increases with age, peaking between 75–79 years old. Fortunately, mortality related to HNC is decreasing (Zhou et al., 2024), contributing to a burgeoning group of HNC survivors with unique survivorship needs.

HNC survivors are distinct from other cancer survivors because they experience disease and treatment-related physical symptoms and bodily changes that are nearly impossible to conceal. HNC survivors struggle with deformations of the face/neck, and impairments in or loss of their ability to speak, hear, smell, or taste; blunted facial expressiveness; skin changes; and excessive/thick saliva production (Tschiesner et al., 2012; Deng et al., 2016; Jiang et al., 2018; Depta et al., 2017). Many of these effects are life-long (Davies-Husband et al., 2018) and, for the majority (∼75%) of survivors, these effects cause embarrassment and body image concerns (Fingeret et al., 2012).

These unique survivorship challenges lead HNC survivors to report, relative to the general population, elevated levels of social isolation (defined as the objective lack of contact with other people) (Smith et al., 2017; van Deudekom et al., 2017; Rhoten et al., 2013), a risk factor for a range of negative health outcomes, including cardiovascular disease, heart attack, stroke, depression, anxiety and mortality. Social isolation occurs in at least 36% of HNC survivors, which is 11% higher than in the general older adult population (National Academies of Sciences E et al., 2020). To prevent social isolation, and the negative health outcomes with which it is associated, identification of HNC survivors at risk for social isolation is critical. Currently, such efforts are hindered by a lack of screening tools that are sufficiently brief to be used in a busy clinical environment, that are designed to assess the prospective risk of developing social isolation, and focus on the unique causes of social isolation in HNC survivors.

Therefore, the purpose of this manuscript is to report on the development of the SIRS (Social Isolation Risk Scale), a brief screening tool for use in the clinic to identify HNC survivors at risk for social isolation and their related risk factors. Future steps seek to psychometrically evaluate the SIRS using a longitudinal design in a sample of 130 HNC survivors assessed between 3 and 9 months post-treatment completion (T1) and again 3 months later (T2).

## Materials and methods

### Overview

The development process draws on Boateng’s Best Practices for Developing and Validating Scales for Health, Social and Behavioral Research (Boateng et al., 2018). Accordingly, we deployed a two-phase development process. The first phase, item generation, used inductive (collecting feedback from HNC experts) and deductive (literature review) methods to generate an exhaustive pool of potential scale items (i.e., reasons why HNC survivors socially isolate, general risk factors for social isolation). The second phase, item refinement, was achieved through a combination of in-person and virtual one-to-one interviews between a study team member and 22 socially isolated HNC survivors.

Phase 1: Item Generation. The inductive methods included twelve, 30-min, virtual, one-to-one discussions between the study PI and individuals with expertise in HNC (medical oncologists, radiation oncologists, surgical oncologists, otolaryngologists, multiple social workers, speech therapist, lymphedema therapist, nurse practitioners, nurses, and physician assistants), scale development, and social isolation, as well as HNC survivors that are part of our advisory board. Additionally, following deductive methods, our study personnel conducted a literature review to identify any additional risk factors for social isolation in this population that were not identified during the one-to-one interviews. For the literature review, the following combination of search terms was used to search the PsycINFO, PubMed, and CINAHL databases on 11 October 2023: “Head and Neck Cancer” AND “social isolation”. Together, these databases allow access to several million articles in the domains of behavioral sciences, mental health, life sciences, biomedical sciences, social sciences, nursing, and allied health professions.

Phase 2: Item Refinement. Demographic and clinical data were collected from N = 22 participants, as follows: age (continuous item), gender (male, female, prefer not to answer), race (White/Caucasian, Black/African American, Asian/Pacific Islander, Other), ethnicity (Hispanic/Latino, Non-Hispanic/Non-Latino, Prefer not to answer), marital status (Married/partnered, single/never married, divorced, widowed), type of head and neck cancer, date of initial diagnosis, and date of last treatment. Next, the participants were asked an open-ended question, “Due to your head and neck cancer diagnosis or treatment, what are the main challenges you encounter that impact socializing?”. Following their qualitative response, participants are given the list of 39 potential SIRS items (which were created during the item generation phase) and asked to check off all that apply with the stem question “due to my head and neck cancer or its treatment, I have challenges with”. Next, participants were asked to rate the checked items considering how much that item impacts their socializing, on a 1-4 scale ranging from “not at all”, to “a little”, to “moderately”, to “very much”. Lastly, participants provided open-ended feedback on any items they would like to add.

### Setting

Phase 1. Item Generation. The individuals who assisted with the item generation phase were all employees of a large healthcare system in New York, with the exception of two individuals, who were employed by a large public university in New York and at a university in California. The head and neck cancer survivors who were part of our advisory board assisted with item generation and were recommended by the head and neck cancer clinician members of our study team.

Phase 2. Item Refinement. The 22 research participants who engaged in the item refinement phase were recruited from the radiation oncology clinic within our large healthcare system. Our research assistant screened the medical record of all survivors coming into the clinic each week, identified those who were potentially eligible, and emailed the list to the clinician for approval to approach. Once approved, the research assistant would visit the clinic to speak with the survivor before or after their appointment, explain the study, solicit interest and confirm eligibility. If interested and eligible, the research assistant would obtain informed consent and administer the study questionnaire. Following completion of this one-time questionnaire, research participants received $20 as compensation for their time. Completion took approximately 30 min.

### Inclusion/exclusion criteria

Phase 1: Item Generation. As the intent of this phase was to capture all possible risk factors for social isolation through many discussions with individuals with various types of expertise, there were no limitations on who could participate.

Phase 2: Item Refinement. In order to participate in the item refinement phase, the following criteria had to be met: 1) a diagnosis of head and neck cancer, as documented in the electronic medical record; 2) have completed treatment for head and neck cancer; 3) have no upcoming cancer treatment; 4) over the age of 18; 5) English-speaking; and 6) score <12 (possible range 0–30) on the 6-item Lubben Social Network Scale, an instrument designed to estimate current social isolation by measuring the number and frequency of social contacts with friends and family members. Lower scores indicate fewer social interactions/contacts. This particular cutoff was selected because scores lower than 12 indicate risk for social isolation on this measure.

### Statistical analyses

Phase 1: Item Generation. During the item generation phase, potential scale items were collected from HNC experts as well as the literature review results, and added to a comprehensive list. The result of this phase was a pool of 39 potential SIRS items.

Phase 2 Item Refinement. Under the guidance of the study biostatistician, frequencies were calculated for each of the 39 potential SIRS items. The visual inspection of the frequency line chart indicated an apparent break-point, which occurred at a frequency of 7 (i.e., the potential SIRS item was endorsed by 7 or more participants). Items with frequencies higher than the break were retained. The retained items were reorganized by how much the item impacts socializing, from most impactful (items with the highest averages on the impact question) to least impactful (items with the lowest averages on the impact question). For items with the same averages on the impact question, they were reorganized from those with the highest to those with the lowest standard deviation. Next, general items were removed based on the rationale that general social isolation risk measurement tools already exist and this tool is intended to be HNC specific.

The remaining 13 items were reorganized so that items with similar content were placed together (i.e., items related to food were grouped together, items related to the senses were grouped together). The stem questions and the Likert-scale response options used during the item refinement phase were retained for the final SIRS.

## Results

Phase 1. Item Generation. The 39 potential SIRS items that were identified during the item generation phase can be found in Table 1. The majority of these 39 items were identified by the HNC experts, while few items (i.e., malodor) were identified during the literature review.

**TABLE 1:** Thirty-nine potential SIRS items created during Item generation and administered to research participants during item refinement.

Speaking or communicating
Drooling, excessive saliva, nasal leakage
Coughing/mucous
Eating or swallowing
Finding suitable food to eat
Use of a feeding tube
Tasting
Lack of appetite
Smelling
Breathing
Fatigue
Hearing
Walking
Incontinence
Disfigurement or facial nerve damage
Sensory changes (pain, numbness, swelling)
Giving off a bad odor (malodor)
Lack of finances
Logistics of going out (cannot drive anymore, do not have someone to drive)
Limited time (too many cancer-related appointments, hospital stays, treatments, therapy)
Preparing to leave the house (putting on makeup, prosthetics, getting dressed, bathing)
People feeling uncomfortable around me
People behaving differently around me
People not contacting me as much as they used to
Feeling embarrassed or self-conscious
Feeling like a burden to family or friends
Talking about my cancer or its treatment
Getting enough help or support from my caregivers
Getting to/from work or staying at work
Weather making my symptoms worse
Prioritizing social relationships
Fear of catching a virus (COVID-19, flu)
Fear of cancer recurrence
Little interest or pleasure in doing things
Feeling down, depressed or hopeless
Safety of surroundings
Substance use
Homelessness
Culture/language

**FIGURE 1:**
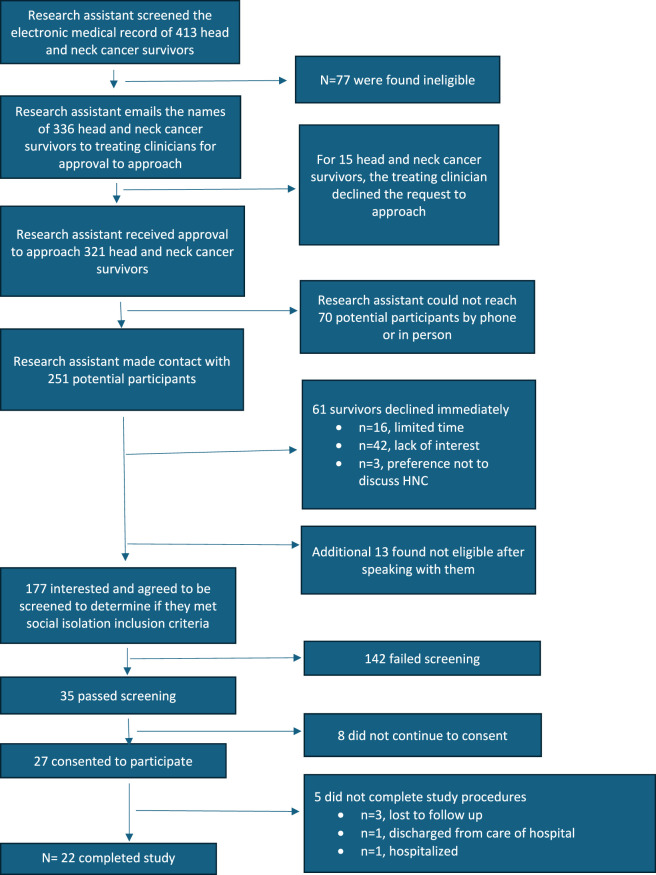
CONSORT diagram.

Phase 2. Item Refinement. During the item refinement phase, we recruited HNC survivors from the clinics of three clinicians (see Figure 1 for CONSORT diagram). Table 2 details the demographics of the 22 participants who completed the study procedures during the item refinement phase. On average, they were 66.41 years old (SD = 9.04 years old), and mostly male (n = 17, 77%). Just over half (n = 12, 54%) identified as White/Caucasian and the majority (n = 18, 82%) were Non-Hispanic/Non-Latino. The most common marital status was married/partnered (n = 12, 54.6%) and the most common type of head and neck cancer was cancer of the tongue (n = 7, 31.9%). Participants were, on average, 3.91 years (SD = 4.79 years) since their initial diagnosis and 3.18 years since completing treatment (SD = 4.81 years).

**TABLE 2:** Demographics of N = 22 participants in the item refinement phase.

Variable	M (SD) or n (%)
Age (years)	66.41 (9.04), range 49–84
Gender Male Female	17 (77.3) 5 (22.7)
Race White/Caucasian Black/African American Asian/Pacific Islander Other	12 (54.6) 4 (18.2) 3 (13.6) 3 (13.6)
Ethnicity Hispanic/Latino Non-Hispanic/Non-Latino	4 (18.2) 18 (81.8)
Marital status Married/partnered Single/never married Divorced Widowed Unknown	12 (54.6) 4 (18.2) 2 (9.1) 3 (13.6) 1 (4.5)
Type of HNC Tongue Tonsil Mouth Larynx Oropharynx Parotid Gland Nasal Cavity/Sinus Nasopharynx Thyroid Gland	7 (31.9) 4 (18.2) 2 (9.1) 2 (9.1) 2 (9.1) 2 (9.1) 1 (4.5) 1 (4.5) 1 (4.5)
Time since initial diagnosis (years)	3.91 (4.79)
Time since completed treatment (years)	3.18 (4.81)

Table 3 shows the 29 items (of the 39 possible items) that were endorsed by at least 7 of the 22 research participants that were part of the item refinement sample, organized from highest to lowest mean (most impact to least impact on socializing). In sum, the items that were both most frequently endorsed (checked off by 7 or more participants) and rated as most impactful on socializing were: fear of catching a virus, challenges prioritizing social relationships, logistics of going out, eating or swallowing, and people not contacting me as much as they used to in the past.

**TABLE 3:** Endorsement frequency of checklist.

Item	n (%)	^b^Mean (SD)
1. Fear of catching a virus^a^	11 (50.0)	3.18 (0.87)
2. Prioritizing social relationships^a^	8 (36.4)	3.13 (0.83)
3. Logistics of going out^a^	7 (31.8)	3.0 (1.15)
4. Eating or swallowing	21 (95.5)	2.9 (1.22)
5. People not contacting me as much as they used to^a^	9 (40.9)	2.89 (1.17)
6. Feeling like a burden to family or friends^a^	8 (36.4)	2.88 (1.25)
7. Lack of finances^a^	7 (31.8)	2.86 (1.46)
8. Little interest or pleasure in doing things^a^	15 (68.2)	2.8 (1.15)
9. People behaving differently around me^a^	8 (36.4)	2.75 (1.04)
10. Sensory changes (pain, numbness, swelling)	11 (50.0)	2.73 (1.35)
11. Fatigue^a^	17 (77.3)	2.71 (1.26)
12. Coughing/Mucous	14 (63.6)	2.64 (1.08)
13. Speaking or communicating	11 (50.0)	2.64 (1.03)
14. Feeling embarrassed or self-conscious^a^	11 (50.0)	2.64 (1.03)
15. People feeling uncomfortable around me^a^	8 (36.4)	2.63 (0.92)
16. Drooling/excessive saliva/nasal leakage	12 (54.5)	2.58 (1.16)
17. Limited time^a^	12 (54.5)	2.58 (1.16)
18. Walking^a^	9 (40.9)	2.56 (1.13)
19. Disfigurement or facial nerve damage	9 (40.9)	2.5 (1.41)
20. Feeling down, depressed, hopeless^a^	14 (63.6)	2.5 (1.09)
21. Lack of appetite	17 (77.3)	2.47 (1.28)
22. Fear of cancer recurrence^a^	14 (63.6)	2.43 (1.22)
23. Tasting	21 (95.5)	2.33 (1.43)
24. Finding suitable food to eat	17 (77.3)	2.29 (1.16)
25. Safety of surroundings^a^	7 (31.8)	2.29 (1.11)
26. Smelling	7 (31.8)	2.29 (0.95)
27. Preparing to leave the house	9 (40.9)	2.11 (1.27)
28. Hearing	10 (45.5)	2 (1.25)
29. Breathing	7 (31.8)	2 (0.82)

^a^ Superscript a indicates the item was removed in the final SIRS.

^b^ Scale ranges from 1–4 (1 = not at all, 2 = a little, 3 = moderately, 4 = very much) in response to the prompt: rank the items that were checked off considering how much that item impacts their socializing.

In response to the open-ended questions “due to your head and neck cancer diagnosis or treatment, what are the main challenges you encounter that impact socializing?” and “provide open-ended feedback on any items you would like to add”, participants expanded on the items in the checklist. Most comments were in reference to difficulty with eating and speaking. Other comments focused on when, within the cancer trajectory, was the most difficult period for socializing, such as around the time of surgery or right after radiation. Others indicated the types of social activities that were impacted by HNC and its treatment, most notably going out to eat with friends or family.

In terms of new areas that were not already mentioned in the checklist, some participants stated that having their teeth extracted greatly impacted their socializing. One participant said that he had to get half of his teeth removed prior to starting treatment, which left him with a lisp. Another participant corroborated this, adding “I had no teeth so I was not going. I did not want people discussing me. I went somewhere with 10 people and they were discussing me. Using a feeding tube was embarrassing. They wanted to make me the topic of conversation.”

Table 4 shows the final SIRS. To arrive at Table 4, our team reviewed Table 3 and removed 16 items that were not specific to HNC (in Table 3, these 16 items are indicated with a superscript a, ^a^), leaving 13 items. Although we feel these 16 items are important, our team decided it was imperative to remain committed to our initial goal of keeping the focus of the SIRS specifically on HNC-related social isolation risk factors. The remaining 13 items were reorganized so that items similar in content were presented together. Content areas were identified as related to food challenges (items 1–4), challenges with the senses (items 4–6), and respiratory challenges (items 7–9), while items 10–13 did not neatly fit together and were therefore placed at the end of the SIRS. As noted, the stem question and response options were retained from the item generation phase.

**TABLE 4:** Final SIRS. Due to my head and neck cancer or its treatment, I have challenges with (check all that apply).

1	Eating or swallowing
2	Lack of appetite
3	Finding suitable food to eat
4	Tasting
5	Smelling
6	Hearing
7	Breathing
8	Coughing
9	Drooling/excessive saliva/nasal leakage/mucous
10	Disfigurement or facial nerve damage
11	Speaking or communicating
12	Sensory changes (pain, numbing, swelling)
13	Preparing to leave the house (putting on makeup to cover disfigurement, securing prosthetics, etc.)

For each of those items checked, rate 1–4 how much that challenge impacts your socializing (1 = not at all; 2 = a little; 3 = moderately; 4 = very much).

## Discussion

Drawing on Boateng’s Best Practices for Developing and Validating Scales for Health, Social and Behavioral Research, our team engaged in two phases (item generation and item refinement) of scale development, leading to the SIRS. The development process was a joint effort by research personnel, HNC experts, experts in social isolation and scale development, and HNC survivors themselves. It centered on frequent communication and iterative discussion among the study team, and took approximately 9 months to complete. Currently, the SIRS is undergoing psychometric evaluation following a longitudinal study with two time points, assessed 3 months apart, in 130 HNC survivors during their first year of survivorship.

Our team sees great benefit in using the SIRS clinically. If found to be psychometrically sound, the strength of the SIRS is in its scope, as it is able to identify, with only 13 questions and in less than 3 min, not only that an individual is at risk for social isolation, but also which specific risk factors he or she has. In a busy clinical setting where a clinician only has minutes with each survivor, this self-administered tool can be used while the patient waits to be seen, and provides a wealth of information about one’s potential for social challenges, which lay at the heart of a range of negative mental health issues. Related, the SIRS has an important place in research settings, as it can identify a sub-group of HNC survivors who are at risk for social isolation and prevention efforts can be delivered. This predictive risk factor-oriented focus differs from the current literature in two ways. First, whereas the SIRS aims to identify risk and prevent social isolation, many current measures in this area aim to identify current social isolation and intervene to reduce it. Second, the SIRS addresses the causal mechanism (social isolation) of negative outcomes, which contrasts with prior studies of HNC survivorship that focus largely on identifying and treating the negative health outcomes themselves (loneliness, depression, anxiety, etc.). Given the complexity and cost of treating these negative health outcomes once they have developed, a risk-oriented preventative approach is more effective and efficient, while also greatly lessening human suffering.

This manuscript reports on a study focused on social isolation, a health problem that has recently emerged as a critical, global public health crisis. The importance of identifying risk for and targeting social isolation in vulnerable populations in this post-pandemic period cannot be underscored. Social isolation was recognized by the 2023 U.S. Surgeon General Dr. Vivek Murthy as a major public health epidemic. Dr. Murthy recognized the immediate dangers of social isolation and developed an advisory on the healing effects of social connection to garner attention for this significant public health challenge. Worldwide, other countries have followed suit, as both Japan and the United Kingdom appointed ministers of social isolation and loneliness as government, cabinet-level positions. These decisions point to the public’s recognition of social isolation as a major health problem.

In addition to addressing a global health crisis, this manuscript is important because it puts into practice the iterative process of scale development outlined in Boateng’s Best Practices. As such, this paper serves as a model on how to develop social isolation risk identification tools for other vulnerable cancer populations in which social functioning is impeded by the illness or treatment (e.g., skin, breast, colon), exponentially expanding the positive impact of this work.

### Study strengths and limitations

Our study has many strengths. One strength of this study is its reliance on feedback from experts in HNC with various perspectives. During Phases 1 and 2, our team obtained input from a comprehensive group that ranged from medical oncologists to HNC survivors themselves. Nearly every individual who has a perspective on HNC was included. A second strength of this study is in the diversity of the research participants. Recruitment occurred at a large healthcare system, which allowed our team to achieve some diversity in gender (77% male, 23% female), race (54% White/Caucasian, 18% Black/African American, 13% Asian/Pacific Islander), and ethnicity (82% Non-Hispanic, 4% Hispanic/Latino). As with any study, we also experienced limitations in this research. Recruitment with a survivor population can be difficult given that this group is not as physically or mentally tied to the clinic setting as they are during their treatment, leading to many potential participants declining due to lack of interest or limited time. In other cases, our study team was not able to make contact via telephone or in person with potential participants. In addition to making recruitment difficult, this also suggests that the feedback we have is from a specific subgroup of HNC survivors who are ready and willing to participate in research, and this may not be representative of all HNC survivors.

## Conclusion

In sum, the SIRS was developed through two phases (item generation and item refinement) as a brief screening tool to identify HNC survivors at risk for social isolation. The theory-driven process of scale development described herein is an example of a research process that integrates perspectives of both the clinical and patient members of the target audience. This step was the first in a program of research in the field of scale development. Future work aims to psychometrically evaluate the SIRS, assess the implementation of the SIRS in a clinic setting, and use the SIRS as the risk identification tool in a randomized controlled trial evaluating targeted versus general social isolation prevention strategies.

## Data Availability

The raw data supporting the conclusions of this article will be made available by the authors, without undue reservation.
